# Effects of Industrial Processing on Pesticide Multiresidues Transfer from Raw Tomatoes to Processed Products

**DOI:** 10.3390/foods9101497

**Published:** 2020-10-19

**Authors:** Francesco Corrias, Alessandro Atzei, Carla Lai, Fabrizio Dedola, Enrico Ibba, Gianluca Zedda, Francesca Canu, Alberto Angioni

**Affiliations:** 1 Department of Life and Environmental Science, Food Toxicology Unit, University of Cagliari, University Campus of Monserrato, SS 554, 09042 Cagliari, Italy; francesco.corrias@unica.it (F.C.); alessandro.atzei@unica.it (A.A.); carla.lai82@googlemail.com (C.L.); francescacanu1996@gmail.com (F.C.); 2 Agricultural Research Agency of Sardinia (AGRIS), Service of Environmental Studies, Crop Protection and Production Quality, Bonassai SS 291 km 18,6, 07100 Sassari, Italy; fdedola@agrisricerca.it (F.D.); eibba@ahrisricerca.it (E.I.); gzedda@agrisricerca.it (G.Z.)

**Keywords:** industrial processing, raw tomato, LC-MS/MS, pesticide residues

## Abstract

Pesticides are broadly used to improve food safety, although they can lead to adverse health effects on consumers. Various food processing approaches, at the industrial or domestic level, have been found to highly reduce the amount of pesticide residues in most food materials. In this work, samples of raw tomatoes were collected directly from the field and processed at the industrial level to produce purée, triple concentrated paste, fine pulp, and diced tomatoes. A multiresidue method based on a modified QuEChERS (Quick, Easy, Cheap, Effective, Rugged e Safe) sample preparation, followed by liquid chromatography-tandem mass spectrometry analysis (LC-MS/MS) for the assessment of 116 pesticides residues, was used. The analytical method has been validated according to SANTE indications. The recovery yields ranged from 75.5% to 115.3%, repeatability (RSDr) ranged from 3.4% to 18.3%, while reproducibility (RSD_wR_) ranged from 5.4% to 19.8%. The limit of quantifications (LOQs) ranged from 2.35 µg kg^−1^ for benthiavalicarb to 6.49 µg kg^−1^ for allethrin. A total of 159 raw tomato samples were collected from the field. The analysis showed the presence of 46 pesticides with azoxystrobin and chlorantraniliprole the most represented. On the other hand, all industrially processed samples showed values ≤ LOD, confirming that post-harvest processes can lead to a decrease in pesticide residues from agricultural commodities.

## 1. Introduction

Tomato (*Solanum lycopersicum* L.) is one of the most important horticultural crops in the world. It belongs to the Solanaceae family, which includes potatoes, peppers, and eggplants [1]. Its botanical origin is not very clear, even if the most qualified thesis places the origin in the narrow band between the Andes mountain ranges and the Pacific coast of western South America, being domesticated in Mexico and spreading in Europe through the Spanish domination [2]. Italy, the United States, and China cover 60% of the entire market. Italy, which accounts for 13% of total world production, represents the second processing country after the USA. With a yield of 4.8 million tons of tomatoes for the processing industry in 2018, it remains Europe’s most important producer followed by Spain and Portugal [3,4]. Tomato is the principal Italian vegetable species, with 80% for industrial processing [5]. In Sardinia, 500,000 quintals of industrial tomatoes from 558 ha were cultivated in 2019 (60% Medio Campidano, 40% Oristano province). Among the most important pests of the open-field tomato, we can count late blight (*Phytophthora infestans*), tomato russet mite (*Aculops lycopersici*), and a particularly harmful and highly destructive tomato moth (*Tuta absoluta*). Integrated pest management (IPM) was the most widespread approach used in pest control by the farmers checked during this survey [6]. Regulation EC No. 396/2005, as amended [7], establishes community provision on maximum residues levels (MRLs). The National Action Plan (PAN) for the sustainable use of plant protection products (PPPs) in Italy has authorized 159 pesticides for the use on tomatoes. PPPs were classified in fungicides (68), insecticides (54), nematocides (13), acaricides (12), herbicide (16), plant growth regulators (4), snail killers (2), and pheromones (2) [8,9]. Consumer concerns about pesticide residues in food are requesting to avoid them as much as possible [10,11]. Although pesticide treatments have been conducted in good agricultural practice (GAP), there is a real possibility that these compounds may release detectable residues even if below the MRLs.

Moreover, treatments with different pesticides can lead to multiresidues contamination of raw tomatoes and can transfer pesticide residues during the processing step from the starting material to the final product [12,13]. Modern multistep and complex food processing approaches significantly decrease the pesticide residues in fruits and vegetables at the industry levels [14]. Pesticide residue levels during processing can be influenced by thermal degradation, evaporation, and co-distillation (blanching, steaming and boiling), dissolving (water or solvents washing), mechanical removal (peeling of vegetable skin) hydrolysis, photolysis, volatilization, and changes in pesticide residue levels due to weight changes [15,16,17,18,19]. Moreover, different LC-MS/MS or GC-MS/MS multiresidue methods for pesticide residue analysis of tomatoes [20,21,22,23,24,25,26,27] and their amount after household processing, concerning washing and peeling [28,29,30,31,32,33], are reported in the literature. Kwon et al. reported both a decrease of non-systemic pesticides by washing and peeling processes and an increase of some systemic pesticide residues in household processed tomatoes due to tomatoe water evaporation during the boiling process [34]. Otherwise, Al-Taher et al. reported a significant decrease of nine pesticides spiked on tomatoes after washing at different temperatures [35]. However, no specific paper can be found analyzing the effect of an entire industrial process on the transfer of pesticide residues from raw tomatoes on the sauce and other processing products. This paper reports a comprehensive study on pesticide residues contamination after the industrial processing of raw tomatoes collected in the field to produce purée, triple concentrated paste, fine pulp, and diced tomatoes. For the analytical determination of pesticide residues on processed commodities and raw tomatoes, a UHPLC-MS/MS multiresidues method with a modified QuEChERS extraction was set up and validated [36].

## 2. Materials and Methods

### 2.1. Samples Collection and Processing

Trials were carried out in an area of 558 ha located in the province of Oristano and Middle Campidano in Sardinia in August 2019. 159 fields ranging from 10 to 1 Ha in size, with seven cultivars (Taylor 28.9%, Creso 19.5%, Dask 21.4%, Docet 19.5%, Datterino 1.3%, Rapidus 1.9%, and undefined cultivar 7.5%) were selected. Raw tomato samples from the field were collected in 15 kg boxes randomly tacking into account plant density and spacing. After that, the boxes were merged, and 1 kg ha^−1^ of raw tomatoes samples was transported to the laboratory. Ten industrial samples for each typology (purée, triple concentrated paste, fine pulp, and diced) were obtained from 5 batches of processing during the working month. Field treatments followed the integrated production strategy in Sardinia for tomatoes with some modification made by the processing factory. Specialized technicians supervised plant protection plans to use, among the authorized pesticides, those with the shortest pre-harvest interval, the lowest toxicity, and the minimum environmental persistence [6].

### 2.2. Chemicals and Reagents

Active ingredients used for qualitative and quantitative analysis were certified analytical standards (≥99.5% purity) from Dr Ehrenstorfer (Lab service Analitica, Milan, Italy) Acetonitrile (ACN), and methanol (MeOH) were LC/MS grade solvents (Sigma Aldrich, Milan, Italy). Formic acid was reagent grade (>95%, Honeywell, Sigma Aldrich), ammonium formate solution 5 M (0.315 g mL^−1^) (G1946-85021, Agilent Technologies). QuEChERS reagents were: Part No.: 5982-6650, 4 g MgSO, 4.1 g NaCl, 1 g trisodium citrate dihydrate, 0.5 g disodium hydrogen citrate sesquihydrate (En Method 15662, Agilent Technologies, Milan, Italy); Part No.: 5982-5056, 150 mg PSA, 900 mg MgSO_4_ (EN Method, fruit and vegetable, Agilent Technologies, Milan, Italy).

MilliQ water with a conductivity less than 18.2 MΩ was obtained from an integrated Millipore purification system (MilliQ integral, Merck, Milan, Italy). The stock solution of pesticide standards (~1000 mg L^−1^) was prepared by weighing about 10 mg of pesticide in a 10 mL volumetric flask, filling up to volume with ACN. Mixed multiresidue pesticide intermediate solution was prepared at 5 mg L^−1^ in ACN. The working solutions were prepared daily by diluting the stock solutions with eluent mixture (MeOH + H_2_O at 0.1% formic acid and 0.5 mM ammonium formate).

### 2.3. Sample Preparation

Tomatoes samples were collected at commercial ripening, brought to the laboratory, chopped, and homogenized with a semi-industrial blender. Industrial samples were collected directly from the processing plant, brought to the laboratory, and blended in a 600 W stainless steel food blender (Girmi, Rimini, Italy). 10 g of homogenized sample were weighed in a 50 mL test tube plus 10 mL of ACN and agitated in the vortex (Reax Top, Heidolph, Germany) for 1 min. After that, 6.5 g of QuEChERS salts (Part No.: 5982-6650) were added, and the test tube was agitated 2 min in the vortex and 15 min in a rotatory shaker. The sample was centrifuged for 5 min at 4000 RPMs and 10 °C (Centrifuge 5810 R, Eppendorf AG 22331 Hamburg). Then, 6 mL of the supernatant were recovered and transferred to a 15 mL test tube containing 1 g of the second QuEChERS salts (Part No.: 5982-5056, Agilent, Milan, Italy). The tube was agitated in vortex for 2 min, and in a rotatory shaker for 15 min, the solution was centrifuged for 5 min at 4000 RPMs at 10 °C, and the organic solution was filtered at 0.45 µm (PTFE, Thermo Scientific, Waltham, MA, USA) and transferred to a 1.8 mL vial for LC-MS/MS analysis.

### 2.4. UHPLC-MS/MS Analysis

A UHPLC Agilent 1290 Infinity II LC coupled with an Agilent 6470 Triple Quad LC-MS/MS mass detector with a MassHunter ChemStation, was used. The column was a ZORBAX Eclipse Plus C18 (2.1 × 150 mm, 1–8 μm). A binary gradient, H_2_O 5 mM in ammonium formate + 0.1% formic acid (A) and methanol 5 mM in ammonium formate + 0.1% formic acid (B) was set as follows: T = 0 A 95%, T = 50 sec A 95%, T = 3.50 min A 60%, T = 17 min A 2%, 10 min of post-run A 95%. The total duration of the run was 27 min, the flow was 0.3 mL/min, with 2 μL of sample volume injected in positive mode. Mass detector gas and sheath-gas were set at 350 °C, gas flow 10 L min^−1^, sheath-gas flow 12 L min^−1^, nebulizer 30 psi, positive capillary 4000 V, dynamic MRM (Supplementary Table S1). 

### 2.5. Method Validation

The analytical method has been validated according to SANTE Guidelines assessing linearity, selectivity, precision, method detection limits (LOD), method quantification limits (LOQ), accuracy in terms of recovery, uncertainty, and matrix effect [37]. Since no blank certified control sample was available on the market, a control field was set up on a reliable farm. The field was subject to organic farming rules, and the tomatoes produced were processed in the laboratory to prepare purée, triple concentrated paste, fine pulp, and diced tomatoes, which have been used as a control matrix sample for method validation. Six blank control samples for each of the five matrices were spiked with the mixed multiresidue standard at 5xLOQ and analyzed in one day for repeatability (RSDr, intraday *n* = 30). In comparison, reproducibility (RSD_wR_) was calculated by the analysis of two samples for each matrix in six separate days (*n* = 60). Each sample belonged to an independent experiment. Recovery assays were carried out, fortifying at LOQ, and 10xLOQ, blank control, with the mixed multiresidue pesticide standard, and left standing for 30 min. Three replicates samples of each concentration were analyzed for each matrix (*n* = 30), as reported above. Recovery results were analyzed using matrix control standard calibration curves. The instrumental sequence was conducted according to SANTE indications. The matrix effect was evaluated by comparing the analytical responses of the active ingredients in ACN + 0.1% formic acid with those prepared with blank control matrix extracts. Linearity was assessed by analyzing five standard calibration curves performed in triplicate, both in solvent and blank control matrix extracts. It was admitted as acceptable when the coefficient of determination was above 0.990. Selectivity was assessed comparing extracts from control matrices with those spiked at the LOQ value. The absence of peaks at the retention times of the a.i. was a criterion for confirmation method selectivity. The expanded measurement uncertainty (U), a quantitative parameter of the reliability of the analytical method, was calculated by multiplying the combined uncertainty (u′) by a coverage factor k = 2, to accomplish a level of confidence of 95%, using the following Equations:

u′ = u′(bias)^2^ + u′(precision)^2^; (1)

U = k × u′(2)

The instrument LOD and LOQ were calculated as three and ten times the signal to noise ratio (S/N) [7,38].

### 2.6. Industrial Processing

Tomatoes samples were mechanically collected from the field in 350 kg bins and brought to the industrial plant within three hours. Then, the tomatoes were left to stand shortly before being subjected to washing and visual selection to reject any tomatoes that are immature, over-ripe, rotten, or damaged. The selected tomatoes were subjected to a blanching step to facilitate skin removal in the subsequent peeling stage. After that, tomatoes followed two different production lines. The first led to purée, triple concentrated paste, fine pulp, and the second to diced tomatoes.

Steps of refilling, acidic adjustment, and concentration (only for triple concentrated paste), packing and sealing, followed by pasteurization and cooling were carried out before storing and marketing.

## 3. Results and Discussion

### 3.1. Validation Method

The experimental design has been planned to evaluate pesticide residues behavior during the industrial processing after harvest. Raw tomatoes, purée, triple concentrated paste, fine pulp, and diced tomatoes have been analyzed for pesticide residues detection by using the multiresidue LC-MS/MS-MRM method previously validated. The proposed MRM method allowed the analysis of 116 pesticides, 85 of which authorized on tomatoes (Supplementary Table S1).

**Figure 1:**
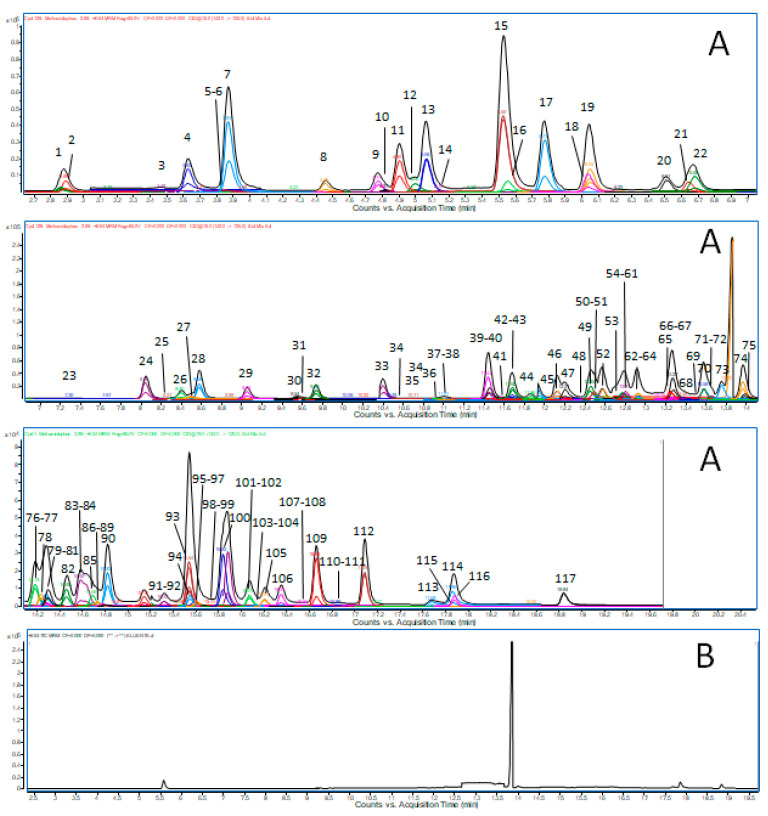
MRM condensed chromatogram at LOQ value of the 116 pesticides analyzed in the present study (**A**), and blank matrix (**B**).

Any difference was detected comparing calibration curves prepared in pure solvents and blank matrix. Therefore, multistandard calibration curves were prepared at five points with minimum and maximum values at LOQ and 100xLOQ in blank control matrix extracts showing correlation coefficients (r^2^) ranging from 0.9959 to 1.0000 and RSD% max 8.65%. Linearity was above the condition set for method validation (Table 1). No interfering peaks were detected in the chromatographic range of interest, and no further cleanup was necessary, showing a reasonable specificity of the method (Figure 1).

Accuracy data provided by recovery experiments from 3 replicate for each matrix for a total of 15 experiments for each concentration tested (Table 1), ranged from 76.6 to 115.3% at LOQ level, and from 75.5 to 109.5% at 10xLOQ, according to SANTE principles, with minimum and maximum coefficient of variability ranging from 0.1 to 19.6% (Table 1). The average value of all recoveries was 94.6% ± 0.09%, which can be considered a good result. Repeatability (RSDr; *n* = 30) and within laboratory reproducibility (RSDwR; *n* = 60) showed good results below 19.1%. Maximum and minimum RSD% were 18.3% and 3.4% in RSDr, and 19.4% and 5.4% in RSDwR (Table 1), with an average value of 11.11% ± 33.6%. According to average recoveries and RSDwR, expanded uncertainty (U) for all pesticides was below 50% of the default values for both spiking levels. Moreover, the method showed good robustness and could be used for the analysis of the studied pesticides in raw and processed tomatoes (Table 1). The instrument limits of quantification (LOQs) and of determination (LODs), calculated as 10-fold and 3-fold the signal-to-noise ratio, were far below the MRLs set by the European Community (Table 1), with LOQ values ranging from 2.35 µg kg^−1^ for benthiavalicarb to 6.49 µg kg^−1^ for allethrin.

### 3.2. Analysis of Raw and Processed Tomatoes

A total of 159 samples were collected from the fields; the analysis of raw tomatoes allowed the identification of 46 pesticides among the 116 searched with the above MRM method. The cultivar Dask and Creso were the most polluted, accounting for 36 residues. A total of 1390 residues have been found spread in all samples. Azoxystrobin (141 times), dimethomorph (106), and chlorantraniliprole (102) were the most frequent in raw tomatoes. These pesticides showed the highest levels of residues, followed by fenarimol (97), spinosyn A (83), and emamectin benzoate (72) (Table 2). All pesticides showed residues values at harvest far below the maximum residue levels (MRLs) established in the EU for tomatoes. In 83.2% of the analysis, the residues were below the LOQ of the method and were not quantifiable. Carbendazim, atrazine-desethyl, carbofuran, phosalone and fenarimol are non-authorized in tomatoes and were found in raw tomatoes, all at levels below the LOQ of the method (Table 2). The presence of these residues could be related to soil or water polluted from the previous crops, grown in the same field. Multiresidue pollution has been registered in many samples, with a maximum number of 22 pesticide residues found in a sample of the cultivar Creso. 35% of the samples showed pesticide residues ≤5, 31% between five and 10, 22% between 10 and 15, while only 12% showed more than 16 pesticide residues. 10 samples from 5 different batches for each processing technology were analyzed in triplicate (600 samples), spread throughout the month of the industrial production to cover all the samples collected in the field, and sent for processing. The analysis of the processed tomatoes showed no residues detectable above the LOD of the method (Table 2). Different papers deal with the decrease of pesticide residues after tomato processing, and this fact can be related to different causes. Tomato acidity is similar to the must during winemaking and can have a degrading effect on some compounds [39]. The peeling of tomato during industrial processing can remove pesticide residues solubilized in the skin epicuticular waxes [28,40].

Pesticides, especially those with low penetration ability, can be removed with reasonable efficiency by washing raw tomatoes before processing in the industrial plant. The effectiveness of this step could depend on pesticide solubility in water or in different chemical solvents [19,28]. Reiler et al. reported the analysis of six organochlorines and five organophosphates in raw tomatoes and after soft laboratory processing. The results showed a significant decrease of the residues after washing and peeling [41]. However, other papers reported a minimal removal effect of washing with water on pesticide residues, regardless of the O/W partition coefficients [42,43]. Moreover, the impact of the industrial process on pesticide residues cannot be compared to household cleaning treatment and model tests in the laboratory. In addition to the washing and peeling process, two main necessary technological operations can lead to residue decrease in tomato factories. The dilution effect, and heating over 100 °C with pressure values below 1 atm used to obtain the tomato juice, which could cause pesticide extraction phenomena in the vapor stream. In particular, the industrial practice contemplates the processing of many batches from the different fields at the same time, thus diluting the possible pollution of raw tomatoes. As can be evinced from the present paper, the samples collected in the field have a heterogeneous residue composition.

Nevertheless, the high volume of tomatoes worked at industrial levels could bring the level of residues in final products to analytical zero. In contrast to what reported in a previous article [34], this phenomenon also occurs during the production of purée and triple concentrate paste. The legal limit for food intended for infants and young children is set at a level equal or close to the limit of quantification; in general, a default MRL of 0.01 mg/kg is applicable unless lower legal limits for the residue levels are defined in Directives 2006/125/EC and 2006/141/EC [44,45]. For this reason, the certificates of the analysis indicate the presence of the pesticide only if it exceeds this level.

## 4. Conclusions

This paper reported the first study on the behaviour of pesticide residues on tomatoes from field contamination to industrially processed products. The presented LC-MS/MS method was developed and validated for the analysis of 116 pesticide residues in tomatoes and their processing products. Moreover, it showed, on average, an LOQ half the established value and LOD 10 times lower, which allows us to quantify pesticide residues at low levels and detect traces in the nanogram range. The above-validated method allowed us to analyze 759 samples from raw and processed tomatoes. Raw tomatoes showed the presence of only 46 pesticides with values always below the MRL and 83.2% below the LOQ. Processed products showed no pesticide residues. The industrial processing coupled with the dilution effect allowed us to decrease pesticide residues found in the raw material below the LOD. Therefore, considering the results of the present paper, when good agriculture practices (GAP) are applied in the field, the final products of the tomato supply chain can reach residue values lower than those established for baby food, accomplishing better human and environmental safety results.

## Figures and Tables

**Table 1:** Linearities, curves, LODs and LOQs and method validation parameters for the analysis of 116 target a.is. in tomato in LC-MS/MS.

Pesticide	Linearity	Linear Regression Equation	R^2^ ± RSD%	MRL	LOD	LOQ	Apparent Recovery (%, *n* = 15)		RSD_r_ (5×LOQ)	RSD_wR_ (5×LOQ)	U *
	(g kg^−1^)			(mg kg^−1^)	(g kg^−1^)	(g kg^−1^)	LOQ	10xLOQ	*n* = 30	*n* = 60	
Cyromazine	LOQ–411	y = 2741924x + 8425	0.9988 ± 0.07	0.60	1.37	4.11	115.3 ± 4.9	108.8 ± 1.7	11.2	12.4	29.4
Methamidophos	LOQ–410	y = 4887931x + 21107	0.9989 ± 0.06	0.01 *	1.37	4.10	105.4 ± 14.3	93.1 ± 10.8	9.0	12.9	38.3
Acephate	LOQ–435	y = 375881x − 144	1.0000 ± 0.10	0.01 *	1.45	4.35	96.5 ± 13.3	90.6 ± 19.4	12.0	17.1	39.8
Formetanate	LOQ–413	y = 14913520x − 5868	1.0000 ± 0.01	0.30	1.38	4.13	84.5 ± 1.9	84.4 ± 9.3	8.6	16.9	33.6
Pymetrozine	LOQ–473	y = 696973x + 6250	0.9959 ± 0.08	0.50	1.58	4.73	101.3 ± 6.7	98.2 ± 5.8	13.9	14.0	16.5
Omethoate	LOQ–621	y = 32351x + 372	0.9987 ± 0.07	0.01 *	2.07	6.21	87.4 ± 7.5	85.1 ± 5.9	7.5	15.1	31.8
Propamocarb	LOQ–417	y = 43247201x − 8829	0.9998 ± 0.18	4.00	1.39	4.17	99.6 ± 6.7	98.5 ± 2.7	13.5	12.9	14.3
Oxamyl	LOQ–401	y = 5159083x − 5579	1.0000 ± 0.01	0.01	1.34	4.01	94.8 ± 11.1	80.9 ± 0.3	5.3	8.1	38.6
Methomyl	LOQ–410	y = 5917725x + 7746	0.9997 ± 0.14	0.01	1.37	4.10	111.8 ± 10.4	107.2 ± 2.3	9.6	10.3	31.0
Flonicamid	LOQ–394	y = 784761x + 1544	0.9998 ± 0.13	0.50	1.31	3.94	95.5 ± 9.6	91.0 ± 12.9	15.4	11.4	29.4
Thiamethoxam	LOQ–524	y = 11189678x + 71281	0.9986 ± 0.08	0.20	1.75	5.24	107.8 ± 8.3	104.5 ± 2.5	7.4	9.8	22.6
Carbendazim	LOQ–400	y = 5329930x + 3082	1.0000 ± 0.01	0.30 *	1.33	4.00	76.6 ± 1.7	83.7 ± 4.1	8.1	12.8	43.2
Monocrotophos	LOQ–414	y = 17644763x + 44731	0.9993 ± 0.11	0.01 *	1.38	4.14	96.4 ± 11.1	79.8 ± 7.4	6.5	10.7	41.8
Chlordimeform	LOQ–396	y = 853728x − 1979	0.9998 ± 0.01	-	1.32	3.96	85.6 ± 7.4	75.5 ± 0.7	14.2	11.4	43.1
Cypermethrin	LOQ–518	y = 5132597x + 20901	0.9979 ± 0.12	0.50	1.73	5.18	86.6 ± 4.1	86.3 ± 7.4	12.6	11.8	29.7
Imidacloprid	LOQ–321	y = 5358564x + 20834	0.9990 ± 0.20	0.50	1.07	3.21	107.4 ± 13.7	107.0 ± 1.2	4.2	6.7	32.1
Methiocarb	LOQ–387	y = 31899495x + 73553	0.9994 ± 0.16	0.20	1.29	3.87	76.9 ± 1.9	80.0 ± 5.2	5.6	9.0	44.7
Dimethoate	LOQ–407	y = 9144917x + 45650	0.9984 ± 0.08	0.02	1.36	4.07	105.3 ± 5.0	96.7 ± 2.7	12.3	8.4	17.7
Acetamiprid	LOQ–398	y = 13742437x + 39503	0.9992 ± 0.79	0.50	1.33	3.98	98.1 ± 8.7	88.1 ± 1.8	6.1	10.7	26.7
Cymoxanil	LOQ–435	y = 7081921x + 5097	0.9998 ± 0.00	0.40	1.45	4.35	99.1 ± 5.8	88.6 ± 5.8	11.4	14.9	24.1
Thiacloprid	LOQ–439	y = 4714164x + 21318	0.9988 ± 0.55	0.50	1.46	4.39	111.8 ± 5.7	106.6 ± 1.2	11.2	12.8	24.4
Atrazine-desethyl	LOQ–430	y = 8730484x + 17192	0.9996 ± 0.03	-	1.43	4.30	102.0 ± 5.2	93.5 ± 3.8	8.4	11.5	18.0
Aldicarb	LOQ–458	y = 22774x − 3	0.9989 ± 0.04	0.02 *	1.53	4.58	99.8 ± 0.2	99.9 ± 0.2	10.0	8.1	10.6
Pirimicarb	LOQ–410	y = 30848679x + 25212	0.9994 ± 0.02	0.50	1.37	4.10	109.8 ± 9.6	108.2 ± 1.8	9.5	7.8	27.7
Dichlorvos	LOQ–410	y = 450147x + 175	1.0000 ± 0.01	0.01 *	1.37	4.10	87.2 ± 4.3	81.7 ± 2.0	8.7	17.1	32.6
Thiophanate-methyl	LOQ–406	y = 17268518x − 53036	0.9997 ± 0.32	1.00	1.35	4.06	93.1 ± 10.0	80.2 ± 4.6	18.1	7.9	38.4
Metribuzin	LOQ–427	y = 3761325x + 17279	0.9983 ± 0.74	0.10	1.42	4.27	98.9 ± 10.7	108.4 ± 2.4	5.6	12.4	26.3
Carbofuran	LOQ–416	y = 27252840x + 33458	0.9996 ± 0.01	0.002 *	1.39	4.16	96.3 ± 2.9	94.9 ± 1.5	8.5	7.7	10.7
Carbaryl	LOQ–410	y = 14046644x + 7098	0.9998 ± 0.22	0.01 *	1.37	4.10	110.4 ± 5.0	100.2 ± 2.1	8.4	11.5	22.9
Imazalil	LOQ–431	y = 972258x + 947	0.9999 ± 0.21	0.50	1.44	4.31	78.8 ± 8.2	77.5 ± 2.2	13.0	16.7	46.0
Fosthiazate	LOQ–397	y = 720338x + 1656	0.9997 ± 0.12	0.02	1.32	3.97	97.2 ± 2.1	98.7 ± 0.1	7.5	10.6	6.4
Disulfoton-Sulfoxide	LOQ–471	y = 12702349x + 19964	0.9998 ± 0.32	-	1.57	4.71	110.8 ± 7.0	107.6 ± 1.2	4.6	16.4	24.7
Flutriafol	LOQ–470	y = 3625x + 183	0.9986 ± 0.04	0.80	1.57	4.70	106.9 ± 8.1	104.9 ± 1.0	15.2	12.4	21.0
Metalaxyl	LOQ–390	y = 4371x + 183	0.9986 ± 0.04	0.20	1.30	3.90	85.6 ± 6.9	84.3 ± 13.1	9.5	10.2	36.7
Methidathion	LOQ–424	y = 396329x − 101	0.9994 ± 0.14	0.02 *	1.41	4.24	93.6 ± 11.4	92.9 ± 2.9	10.2	11.1	26.2
Azinphos-methyl	LOQ–464	y = 398216x + 390	0.9998 ± 0.58	0.05 *	1.55	4.64	98.2 ± 9.9	93.6 ± 2.3	8.5	19.4	22.5
Chlorantraniliprole	LOQ–399	y = 585240x + 2	0.9997 ± 0.01	0.60	1.33	3.99	92.1 ± 5.8	89.6 ± 4.5	7.4	15.7	22.2
Pyrimethanil	LOQ–395	y = 1208059x − 4250	0.9991 ± 0.05	1.00	1.32	3.95	109.7 ± 5.5	105.4 ± 0.3	7.4	15.7	20.9
Azoxystrobin	LOQ–431	y = 37027632 + 20546	0.9999 ± 0.31	3.00	1.44	4.31	103.4 ± 5.5	99.7 ± 4.2	6.7	19.8	14.5
Diethofencarb	LOQ–385	y = 17914008x +75801	0.9990 ± 1.21	0.70 *	1.28	3.85	97.0 ± 14.2	83.9 ± 14.6	6.4	15.5	43.4
Propanil	LOQ–392	y = 1172102x − 4083	0.9972 ± 0.09	0.01 *	1.31	3.92	90.9 ± 5.1	91.0 ± 5.8	12.5	13.0	22.0
Fenamidone	LOQ–390	y = 19532597x + 43718	0.9995 ± 1.06	1.00	1.30	3.90	96.5 ± 9.5	103.2 ± 1.3	9.4	14.5	21.5
Diclobutrazol	LOQ–391	y = 145538x + 476	0.9993 ± 0.22	-	1.30	3.91	101.5 ± 0.7	102.0 ± 0.2	8.7	10.3	3.8
Boscalid	LOQ–408	y = 4252887x + 877	1.0000 ± 0.30	3.00	1.36	4.08	104.5 ± 8.6	105.9 ± 0.3	14.1	15.5	20.6
Dimethomorph	LOQ–394	y = 14639124x + 39105	0.9980 ± 0.16	1.00	1.31	3.94	93.8 ± 17.4	97.9 ± 2.5	8.9	8.7	35.5
Mandipropamid	LOQ–455	y = 5148550x − 20963	0.9983 ± 5.08	3.00	1.52	4.55	85.6 ± 11.9	82.1 ± 8.4	5.6	14.8	40.6
Benthiavalicarb	LOQ–235	y = 4160577x + 5366	0.9994 ± 0.97	0.30	0.78	2.35	111.4 ± 5.8	106.8 ± 0.9	9.7	11.5	24.0
Molinate	LOQ–621	y = 47567x − 140	0.9999 ± 0.93	0.01 *	2.07	6.21	92.2 ± 3.1	85.9 ± 4.6	8.1	15.4	24.5
Chloroxuron	LOQ–425	y = 12252052x − 45885	0.9984 ± 0.19	0.01 *	1.42	4.25	105.7 ± 5.7	102.3 ± 0.4	4.5	10.5	15.7
Myclobutanil	LOQ–411	y = 123049x − 122	0.9997 ± 0.07	0.30	1.37	4.11	83.1 ± 6.1	91.8 ± 0.7	13.4	13.7	32.5
Bifenazate	LOQ–399	y = 1008299x + 76836	0.9961 ± 1.07	0.50	1.33	3.99	87.0 ± 14.1	90.8 ± 19.6	5.6	15.5	44.4
Cyproconazole 1	LOQ–410	y = 14106016x + 110236	0.9985 ± 4.29	0.05	1.37	4.10	109.2 ± 7.2	105.7 ± 1.1	10.8	18.6	22.6
Triadimenol	LOQ–495	y = 3828694x + 27482	0.9970 ± 5.79	1.00	1.65	4.95	84.9 ± 13.3	96.4 ± 8.1	8.4	15.7	38.8
Iprovalicarb	LOQ–408	y = 4408560x + 40826	0.9967 ± 0.22	0.70	1.36	4.08	90.6 ± 14.0	102.9 ± 0.4	12.4	13.5	33.7
Fenhexamid	LOQ–436	y = 3699942x + 18108	0.9991 ±1.57	2.00	1.45	4.36	102.8 ± 5.9	82.3 ± 3.6	3.4	18.5	36.8
Azinphos-ethyl	LOQ–391	y = 367433x + 492	0.9997 ± 0.39	0.02 *	1.30	3.91	79.1 ± 9.5	95.7 ± 0.5	7.6	15.4	43.2
Tetraconazole	LOQ–516	y = 9182219x − 30061	0.9993 ± 0.09	0.10	1.72	5.16	106.8 ± 7.4	109.5 ± 0.9	12.1	15.3	22.4
Cyproconazole 2	LOQ–410	y = 5446075x − 17111	0.9994 ± 5.95	0.05	1.37	4.10	105.3 ± 7.5	102.7 ± 1.1	7.7	13.5	18.3
Mepanipyrim	LOQ–407	y = 328904x − 348	0.9991 ± 2.87	1.50	1.36	4.07	96.6 ± 2.1	100.8 ± 0.6	13.3	19.1	8.5
Spirotetramat	LOQ–503	y = 2820842x + 73839	0.9997 ± 0.67	2.00	1.68	5.03	85.6 ± 5.6	98.5 ± 1.7	6.5	18.3	29.9
Flufenacet	LOQ–414	y = 10522988x + 50386	0.9983 ± 8.65	0.05	1.38	4.14	89.9 ± 8.6	89.9 ± 0.4	10.5	14.8	28.4
Ethoprop	LOQ–377	y = 5056608x + 20031	0.9990 ± 2.42	0.02	1.26	3.77	89.5 ± 10.1	88.4 ± 1.2	8.9	10.4	29.2
Bupirimate	LOQ–396	y = 7147770x + 5118	0.9998 ± 0.37	2.00	1.32	3.96	91.8 ± 4.5	81.9 ± 10.2	5.4	18.8	33.1
Cyazofamid	LOQ–405	y = 54070x + 229	0.9992 ± 0.72	0.60	1.35	4.05	101.7 ± 1.0	102.7 ± 0.2	5.6	12.8	5.0
Flusilazole	LOQ–457	y = 9842141x + 54831	0.9982 ± 2.81	0.01 *	1.52	4.57	106.4 ± 8.2	104.1 ± 0.2	5.8	14.9	20.7
Cyprodinil	LOQ–408	y = 2997671x + 13504	0.9992 ± 0.05	1.50	1.36	4.08	93.9 ± 15.1	79.8 ± 1.9	4.6	18.7	45.0
Fenamiphos	LOQ–547	y = 2610314x + 116029	0.9991 ± 0.92	0.04	1.82	5.47	103.9 ± 7.1	100.7 ± 1.2	11.2	15.6	16.5
Iprodione	LOQ–455	y = 82937x + 89	0.9997 ± 0.22	5.00	1.52	4.55	94.4 ± 4.0	96.5 ± 4.2	9.9	15.8	14.0
Aclonifen	LOQ–393	y = 96057x + 89	0.9997 ± 0.22	0.01	1.31	3.93	97.5 ± 1.2	96.2 ± 0.7	12.4	12.5	7.0
Penconazole	LOQ–398	y = 6090424x + 15097	0.9992 ± 0.43	0.10	1.33	3.98	104.6 ± 6.2	102.4 ± 0.1	9.1	11.4	15.2
Tebuconazole	LOQ–401	y = 19544180x + 77610	0.9989 ± 0.56	0.90	1.34	4.01	102.1 ± 2.1	104.4 ± 1.1	7.1	10.4	8.4
Napropamide	LOQ–413	y = 6724980x + 62380	0.9964 ± 0.81	0.10	1.38	4.13	108.4 ± 6.2	104.8 ± 11.1	8.5	13.8	25.6
Benalaxyl	LOQ–481	y = 23788991x + 92142	0.9982 ± 0.19	0.50	1.60	4.81	100.6 ± 4.9	108.6 ± 1.1	8.9	9.4	17.4
Spinosyn A	LOQ–432	y = 1125007x − 3076	0.9997 ± 0.01	0.70	1.44	4.32	77.0 ± 4.1	76.7 ± 0.6	15.5	14.5	46.8
Zoxamide	LOQ–410	y = 5772043x + 12727	0.9994 ± 0.04	0.5	1.37	4.10	92.1 ± 9.4	81.5 ± 4.8	16.5	13.2	35.7
Pyraclostrobin	LOQ–418	y = 13354734x − 5243	1.0000 ± 3.84	0.30	1.39	4.18	94.5 ± 8.2	92.8 ± 3.9	5.8	15.7	21.0
Cyflufenamid	LOQ–408	y = 5945864x + 38431	0.9977 ± 0.65	0.04	1.36	4.08	87.5 ± 17.6	91.1 ± 1.1	17.4	18.5	40.0
Bitertanol	LOQ–406	y = 6031192x + 40609	0.9969 ± 0.80	0.01 *	1.35	4.06	105.1 ± 1.7	102.6 ± 0.9	11.2	14.6	9.8
Clofentezin	LOQ–408	y = 1910622x − 533	0.9999 ± 0.95	0.30	1.36	4.08	83.2 ± 11.4	103.7 ± 3.8	8.9	8.1	42.8
Phosalone	LOQ–578	y = 6702437x + 16186	0.9995 ± 0.02	0.01 *	1.93	5.78	99.6 ± 3.4	94.2 ± 3.1	3.4	12.8	12.8
Metrafenone	LOQ–468	y = 9239760x + 20728	0.9999 ± 0.02	0.40	1.56	4.68	96.4 ± 1.9	101.0 ± 4.9	5.6	8.4	11.4
Difenconazole	LOQ–485	y = 20779327x + 20116	0.9999 ± 0.02	2.00	1.62	4.85	97.4 ± 7.3	95.8 ± 1.8	10.0	9.4	16.3
Chlorpyrifos-methyl	LOQ–414	y = 65820x − 225	0.9999 ± 0.71	0.50	1.38	4.14	91.2 ± 7.9	79.1 ± 2.3	9.1	11.4	37.5
Ametoctradin	LOQ–317	y = 8626247x + 1875	0.9999 ± 0.01	2.00	1.06	3.17	96.1 ± 7.1	92.8 ± 0.4	8.4	18.7	18.2
Spinosyn D	LOQ–432	y = 209477x − 615	0.9997 ± 0.41	0.70	1.44	4.32	92.9 ± 7.6	89.4 ± 4.1	12.7	5.6	23.8
Indoxacarb	LOQ–450	y = 1790071x − 2915	0.9985 ± 0.01	0.50	1.50	4.50	94.1 ± 1.5	82.2 ± 4.1	8.4	18.9	29.5
Cycloate	LOQ–502	y = 598248x − 1864	0.9974 ± 0.15	-	1.67	5.02	101.6 ± 6.7	101.3 ± 0.1	10.1	15.4	13.8
Hexaflumuron	LOQ–419	y = 633012x − 654	0.9977 ± 1.28	-	1.40	4.19	81.8 ± 10.2	81.7 ± 1.3	5.6	14.7	41.0
Trifloxystrobin	LOQ–432	y = 17415017x + 17609	0.9999 ± 0.27	0.70	1.44	4.32	96.7 ± 7.3	94.7 ± 2.0	7.6	8.4	17.1
Quizalofop-ethyl	LOQ–398	y = 3145820x − 5888	0.9998 ± 0.18	0.40	1.33	3.98	100.8 ± 5.7	100.1 ± 1.5	11.3	5.4	11.8
Cycloxydim	LOQ–407	y = 137254x + 708	0.9986 ± 0.01	1.50	1.36	4.07	85.2 ± 7.8	96.3 ± 0.8	14.2	12.1	30.9
Buprofezin	LOQ–465	y = 20507228x + 51039	0.9998 ± 0.89	1.00	1.55	4.65	95.0 ± 8.0	93.9 ± 0.7	8.4	14.0	19.2
Tebufenpyrad	LOQ–401	y = 4341528x + 15573	0.9994 ± 1.27	0.80	1.34	4.01	83.6 ± 6.8	81.7 ± 1.8	9.7	9.4	36.7
Emamectin Benzoate	LOQ–522	y = 3632831x − 14712	0.9998 ± 0.25	0.02	1.74	5.22	98.8 ± 5.2	95.2 ± 3.1	12.4	7.4	13.7
Propaquizafop	LOQ–431	y = 2547342x − 6777	0.9971 ± 0.02	0.05	1.44	4.31	99.6 ± 8.8	100.0 ± 2.5	15.4	16.1	18.1
Metaflumizone	LOQ–410	y = 949238x − 6681	0.9983 ± 0.03	0.60	1.37	4.10	90.1 ± 3.8	75.6 ± 8.6	10.6	13.0	41.8
Oxadiazon	LOQ–403	y = 726169x − 4189	0.9983 ± 0.05	0.05	1.34	4.03	94.3 ± 4.8	88.1 ± 0.4	8.4	8.4	21.4
Allethrin	LOQ–649	y = 439397x − 5650	0.9963 ± 0.22	-	2.16	6.49	87.7 ± 4.3	81.2 ± 0.1	10.1	10.4	32.7
Piperonyl butoxide	LOQ–404	y = 31536094x + 11745	0.9999 ± 0.01	-	1.35	4.04	104.8 ± 5.5	100.9 ± 1.8	5.6	9.7	14.6
Chlorpyriphos	LOQ–395	y = 638457x − 2533	0.9992 ± 0.01	0.01	1.32	3.95	104.5 ± 5.1	109.5 ± 6.0	11.4	7.4	16.1
Hexythiazox	LOQ–358	y = 10457257x − 49960	0.9987 ± 0.01	0.50	1.19	3.58	97.9 ± 5.8	97.1 ± 0.5	14.3	14.1	12.5
Pyriproxyfen	LOQ–418	y = 6633898x − 13776	0.9994 ± 0.02	1.00	1.39	4.18	83.4 ± 4.8	77.7 ± 3.5	15.8	12.8	40.2
Pendimethalin	LOQ–333	y = 1172935x − 2381	0.9999 ± 0.01	0.05	1.11	3.33	84.2 ± 6.3	81.0 ± 4.6	14.2	8.4	36.9
Flufenoxuron	LOQ–391	y = 3953910x + 11436	0.9990 ± 0.48	0.50 *	1.30	3.91	81.9 ± 7.4	79.5 ± 1.5	9.7	9.5	40.7
Propargite	LOQ–382	y = 6439838x + 13171	0.9997 ± 1.64	0.01 *	1.27	3.82	86.8 ± 6.0	81.8 ± 1.2	14.1	14.5	33.5
Lufenuron	LOQ–437	y = 568999x + 552	0.9993 ± 0.09	0.50	1.46	4.37	93.5 ± 5.4	96.3 ± 0.6	7.5	12.3	15.7
Etoxazole	LOQ–516	y = 393488x + 312	0.9998 ± 0.02	0.07	1.72	5.16	88.7 ± 6.6	83.2 ± 2.7	18.3	17.2	31.5
Fenpyroximate(E)	LOQ–460	y = 24740316x + 67928	0.9987 ± 0.41	0.20	1.53	4.60	96.2 ± 8.0	95.2 ± 1.2	10.3	9.3	18.0
Deltamethrin	LOQ–345	y = 212931x + 1529	0.9999 ± 0.01	0.07	1.15	3.45	96.8 ± 5.7	79.9 ± 1.1	4.8	10.3	36.0
Acrinathrin	LOQ–475	y = 48200x − 207	0.9998 ± 0.27	0.10	1.58	4.75	89.9 ± 12.2	88.8 ± 10.7	5.4	8.3	34.6
Pyridaben	LOQ–418	y = 18219756x + 7898	0.9999 ± 0.07	0.30	1.39	4.18	98.6 ± 2.6	88.7 ± 9.2	8.6	13.4	23.2
Tau–Fluvalinate	LOQ–431	y = 17740157x − 22927	0.9999 ± 0.13	0.10	1.44	4.31	85.0 ± 11.4	94.2 ± 3.8	4.5	14.1	34.7
Fenarimol	LOQ–444	y = 167645x + 140	0.9999 ± 0.57	0.02 *	1.48	4.44	84.2 ± 19.4	86.3 ± 10.2	11.1	5.6	48.7
Etofenprox	LOQ–387	y = 5530818x + 37481	0.9981 ± 0.24	1.00	1.29	3.87	92.4 ± 5.6	93.3 ± 0.5	7.2	13.1	18.0
Bifenthrin	LOQ–417	y = 39808x + 128	0.9989 ± 0.58	0.30 *	1.39	4.17	80.4 ± 12.6	76.2 ± 1.4	11.1	8.7	49.2
Famoxadone	LOQ–376	y = 168731x − 107	1.0000 ± 0.07	2.00	1.25	3.76	91.7 ± 10.9	94.4 ± 7.1	8.7	9.8	27.6

* Pesticides not allowed in EU with an MRL on tomatoes.

**Table 2:** Pesticide residues concentration (minimum and maximum μg/kg) in raw tomatoes and processed products analyzed during the survey.

Pesticide	Samples *	Min–Max (Average) (µg kg^−1^)										
	Frequency	Raw Tomatoes							Puree	Triple Concentrate	Pulp	Diced Tomatoes
		Creso (31)	Dask (34)	Datterino (2)	Docet (31)	Rapidus (3)	Taylor (46)	Mixed (12)				
Formetanate	68	<LOQ	<LOQ	<LOQ	<LOQ	<LOQ	<LOQ -20.58 (5.69)	<LOQ	<LOD	<LOD	<LOD	<LOD
Propamocarb	15	<LOQ	<LOQ–26.87 (4.71)	<LOD	<LOQ	<LOD	<LOQ	<LOD	<LOD	<LOD	<LOD	<LOD
Flonicamid	9	<LOQ	<LOQ–10.49 (5.73)	<LOD	<LOD	<LOD	9.55	<LOD	<LOD	<LOD	<LOD	<LOD
Carbendazim *	2	<LOD	<LOD	<LOD	<LOD	<LOD	<LOQ	<LOD	<LOD	<LOD	<LOD	<LOD
Imidacloprid	25	<LOQ	<LOQ	<LOD	<LOQ	<LOQ	<LOQ	<LOD	<LOD	<LOD	<LOD	<LOD
Methiocarb	8	<LOD	<LOQ	<LOD	<LOD	<LOD	<LOQ	<LOD	<LOD	<LOD	<LOD	<LOD
Dimethoate	1	<LOQ	<LOD	<LOD	<LOD	<LOD	<LOD	<LOD	<LOD	<LOD	<LOD	<LOD
Acetamiprid	15	<LOQ	<LOQ	<LOD	<LOQ	<LOQ	<LOD	<LOQ	<LOD	<LOD	<LOD	<LOD
Cymoxanil	1	<LOD	<LOD	<LOD	<LOD	<LOD	6.51	<LOD	<LOD	<LOD	<LOD	<LOD
Thiacloprid	4	<LOQ	<LOD	<LOD	<LOQ	<LOD	<LOD	<LOD	<LOD	<LOD	<LOD	<LOD
Atrazine-desethyl *	1	<LOD	<LOQ	<LOD	<LOD	<LOD	<LOD	<LOD	<LOD	<LOD	<LOD	<LOD
Metribuzin	1	<LOD	<LOQ	<LOD	<LOD	<LOD	<LOD	<LOD	<LOD	<LOD	<LOD	<LOD
Carbofuran *	5	<LOQ	<LOQ	<LOD	<LOD	<LOD	<LOD	<LOQ	<LOD	<LOD	<LOD	<LOD
Chlorantraniliprole	102	<LOQ–111.76 (29.37)	<LOQ–205.19 (44.45)	29.74–40.81 (35.28)	<LOQ–50.95 (16.04)	51.78	<LOQ–139.75 (23.11)	<LOQ–37.10 (22.03)	<LOD	<LOD	<LOD	<LOD
Pyrimethanil	13	<LOQ	<LOQ	<LOQ	<LOQ	<LOQ	<LOQ	<LOQ	<LOD	<LOD	<LOD	<LOD
Azoxystrobin	141	17.99–201.98 (80.80)	<LOQ–32.75 (8.27)	<LOQ	<LOQ	26.96	<LOQ–129.65 (22.00)	7.67–72.16 (31.15)	<LOD	<LOD	<LOD	<LOD
Fenamidone	4	<LOD	<LOQ	<LOQ	<LOD	<LOD	<LOQ	<LOQ	<LOD	<LOD	<LOD	<LOD
Boscalid	15	<LOQ -45.13	7.59–69.62 (38.60)	<LOD	<LOQ	<LOQ	<LOQ–442.23 (112.86)	<LOD	<LOD	<LOD	<LOD	<LOD
Dimethomorph	106	<LOQ	<LOQ–170.19 (31.45)	<LOQ	<LOQ	7.70	27.42–71.13 (47.23)	<LOQ–655.78 (264.91)	<LOD	<LOD	<LOD	<LOD
Iprovalicarb	11	5.80–10.77 (15.60)	<LOD	<LOD	15.59	<LOQ	<LOD	<LOQ	<LOD	<LOD	<LOD	<LOD
Tetraconazole	49	<LOQ	<LOQ	<LOQ	<LOQ	<LOQ	<LOQ–34.21 (18.72)	<LOQ	<LOD	<LOD	<LOD	<LOD
Spirotetramat	67	<LOQ	<LOQ	<LOQ	<LOQ	<LOD	<LOQ	<LOQ	<LOD	<LOD	<LOD	<LOD
Penconazole	4	<LOQ	4.22	<LOD	<LOQ	<LOD	<LOD	<LOD	<LOD	<LOD	<LOD	<LOD
Tebuconazole	21	<LOQ	<LOQ	<LOD	<LOQ	<LOQ	<LOQ	<LOQ	<LOD	<LOD	<LOD	<LOD
Benalaxyl	62	<LOQ	<LOQ	<LOQ	<LOQ	<LOQ	<LOQ	<LOQ	<LOD	<LOD	<LOD	<LOD
Spinosyn A	83	<LOQ–70.61 (11.60)	<LOQ–216.78 (24.54)	<LOQ	<LOQ–39.65 (27.03)	<LOQ	36.79	<LOQ–9.83 (4.51)	<LOD	<LOD	<LOD	<LOD
Zoxamide	5	<LOQ	<LOQ	<LOD	<LOD	<LOQ	<LOD	<LOQ	<LOD	<LOD	<LOD	<LOD
Pyraclostrobin	42	<LOQ -128.01 (22.25)	<LOQ	<LOQ	<LOQ	<LOQ	<LOQ–22.78 (11.50)	<LOQ	<LOD	<LOD	<LOD	<LOD
Clofentezine	1	<LOD	<LOD	<LOD	<LOD	<LOD	<LOQ	<LOD	<LOD	<LOD	<LOD	<LOD
Phosalone *	9	<LOQ	<LOD	<LOQ	<LOQ	<LOD	<LOQ	<LOD	<LOD	<LOD	<LOD	<LOD
Difenconazole	14	<LOQ–54.00 (24.87)	<LOQ	<LOD	<LOD	<LOD	20.79	<LOQ	<LOD	<LOD	<LOD	<LOD
Ametoctradin	60	<LOQ–134.60 (27.30)	<LOQ–606.10 (86.72)	<LOQ	<LOQ	<LOQ	<LOQ	<LOQ	<LOD	<LOD	<LOD	<LOD
Spinosyn D	49	<LOQ–97.36 (38.09)	<LOQ–352.24 (92.88)	<LOD	<LOQ–117.68 (86.79)	<LOD	<LOQ–349.00 (70.46)	10.32–12.62 (11.46)	<LOD	<LOD	<LOD	<LOD
Indoxacarb	4	11.20–17.60 (14.40)	6.25	<LOD	<LOD	<LOD	7.31	<LOD	<LOD	<LOD	<LOD	<LOD
Trifloxystrobin	2	<LOD	<LOQ	<LOD	<LOQ	<LOD	<LOD	<LOD	<LOD	<LOD	<LOD	<LOD
Quizalofop-ethyl	2	<LOQ	<LOD	<LOD	<LOD	<LOD	<LOD	<LOD	<LOD	<LOD	<LOD	<LOD
Emamectin Benzoate	72	<LOQ	<LOQ	<LOQ	<LOQ	<LOQ	<LOQ–7.97 (5.58)	<LOQ–17.95 (6.92)	<LOD	<LOD	<LOD	<LOD
Piperonyl butoxide	59	<LOQ–4.62 (4.62)	<LOQ	<LOQ	<LOQ	<LOQ	<LOQ	<LOQ	<LOD	<LOD	<LOD	<LOD
Chlorpyriphos	14	<LOQ	<LOQ	<LOD	<LOQ	<LOD	<LOQ	<LOD	<LOD	<LOD	<LOD	<LOD
Hexythiazox	38	4.87–18.57 (13.84)	6.18–43.72 (16.65)	9.56	4.87–11.52 (7.12)	<LOD	7.76–23.47 (12.58)	17.89	<LOD	<LOD	<LOD	<LOD
Pyriproxyfen	2	<LOQ	<LOD	<LOD	<LOQ	<LOD	<LOD	<LOD	<LOD	<LOD	<LOD	<LOD
Pendimethalin	1	<LOD	<LOQ	<LOD	<LOD	<LOD	<LOD	<LOD	<LOD	<LOD	<LOD	<LOD
Fenpyroximate(E)	2	<LOD	12.30	<LOD	<LOD	<LOD	<LOQ	<LOD	<LOD	<LOD	<LOD	<LOD
Deltamethrin	26	<LOQ–12.48 (8.02)	<LOQ–7.47 (4.05)		<LOQ–7.11 (5.57)		<LOQ–15.41 (7.19)	4.78	<LOD	<LOD	<LOD	<LOD
Fenarimol *	97	<LOQ	<LOQ	<LOQ	<LOQ	<LOQ	<LOQ	<LOQ	<LOD	<LOD	<LOD	<LOD

* Non authorized pesticide in tomatoes.

## Supplementary Materials

The following are available online at https://www.mdpi.com/2304-8158/9/10/1497/s1, Table S1: Active ingredients, types, and LC-MS/MS-MRM m/z ions used for qualitative and quantitative analysis.

## References

[1] Knapp S., Peralta I.E., Causse M., Giovannoni J., Bouzayen M., Zouine M. (2016). The Tomato (*Solanum lycopersicum* L., Solanaceae) and Its Botanical Relatives. The Tomato Genome (Compendium of Plant Genomes).

[2] OECD (2017). “Tomato (Solanum lycopersicum)”, in Safety Assessment of Transgenic Organisms in the Environment.

[3] FAOSTAT (2017). Food and Agriculture Organization. http://www.fao.org/faostat/en/#data/QC.

[4] Italian Processed Tomato Overview 2018. Gain Reports IT1838, USDA Foreign Agricultural Service, 29/03/2018. https://www.fas.usda.gov/data/italy-italian-processed-tomato-overview-2018.

[5] ISTAT—Production Areas Jointed. http://dati.istat.it/.

[6] (2015). DECRETO N. 501 DECA 11 DEL 18 MARZO. Rules of Integrated Tomato Production of the Sardinia Region. Defense of Tomato in Field and Industry. https://www.sardegnaambiente.it/documenti/1_19_20150320134850.pdf.

[7] REGULATION (EC) NO 396/2005 of the European Parliament and of the Council of 23 February 2005 on Maximum Residue Levels of Pesticides in or on Food and Feed of Plant and Animal Origin and Amending Council Directive 91/414/EEC. Official Journal of the European Union. L 70/1. https://eur-lex.europa.eu/legal-content/EN/TXT/PDF/?uri=CELEX:32005R0396&from=EN.

[8] National Action Plan (PAN) for the Sustainable Use of Plant Protection Products in Italy. https://www.minambiente.it/sites/default/files/archivio/allegati/vari/pubbl_PAN.pdf.

[9] SANTE/E4/VW 10235/2016—Rev. 4, Commission Working Document, on the Evaluation of Data Submitted to Confirm MRLs. Following the Review of Existing MRLs. Brussels. https://ec.europa.eu/food/sites/food/files/plant/docs/pesticides_mrl_guidelines_sanco-10235-2016.pdf.

[10] Krishna V.V., Qaim M. (2008). Consumer attitudes toward GM food and pesticide residues in India. Eur. Rev. Agric. Econ..

[11] Suresh A., Kjha G., Raghav S., Supriya P., Lama A., Punera B., Kumar R., Handral A.R., Sethy J., Gidey R.G. (2015). Food safety concerns of consumers: A case study of pesticide residues on vegetables in Delhi. Eur. Rev. Agric. Econ..

[12] Relyea R.A. (2008). A cocktail of contaminants: How mixtures of pesticides at low concentrations affect aquatic communities. Oecologia.

[13] Pesticide Action Network Pesticide Cocktail in European Food, Brussels, Press Release. https://www.pan-europe.info/press-releases/2019/07/pesticide-cocktails-european-food.

[14] Keikotlhaile B.M., Spanoghe P., Steurbaut W. (2010). Effect of food processing on pesticides residues in fruits and vegetables: A meta-analysis approach. Food Chem. Toxicol..

[15] Dordević T., Durović-Pejčev R. (2016). Food processing as a means for pesticide residue dissipation. Pestic. Phytomed..

[16] Holland P.T., Hamilton D., Ohlin B., Skidmore M.W. (1994). Effects of storage and processing on pesticide residues in plant products. J. Macromol..

[17] Kaushik G., Satya S., Naik S.N. (2009). Food processing a tool to pesticide residue dissipation—A review. Food Res. Int..

[18] Chavarri M.J., Herrera A., Arino A. (2005). The decrease in pesticides in fruit and vegetables during commercial processing. Int. J. Food. Sci. Technol..

[19] Krol W.J., Arsenault T.L., Pylypiw H.M., Mattina M.J.I. (2000). Reduction of pesticide residues on produce by rinsing. J. Agric. Food Chem..

[20] Sannino A., Bolzoni L., Bandini M. (2004). Application of liquid chromatography with electrospray tandem mass spectrometry to the determination of a new generation of pesticides in processed fruits and vegetables. J. Chrom. A.

[21] Silva-Rodríguez A., Acedo-Valenzuela M.I., Diez N.M.M., de la Peña A.M., Galeano-Díaz T. (2012). Multiresidue method for the control of pesticide residues in tomatoes and derived products. Anal. Methods.

[22] Salamzadeh J., Shakoori A., Moradi V. (2018). Occurrence of multiclass pesticide residues in tomato samples collected from different markets of Iran. J. Environ. Health Sci. Eng..

[23] Sartain M., Fandino A., Glauner T. (2015). Routine Multiresidue Pesticide Analysis using the Agilent 6470 Triple Quadrupole Mass Spectrometer. Agilent Application Note.

[24] Jahanmard E., Ansari F., Feizi M. (2016). Evaluation of QuEChERS sample preparation and GC mass spectrometry method for the determination of 15 pesticide residues in tomatoes used in salad production plants. Iran J. Public Health.

[25] Golge O., Kabak B. (2015). Evaluation of QuEChERS sample preparation and liquid chromatography–triple-quadrupole mass spectrometry method for the determination of 109 pesticide residues in tomatoes. Food Chem..

[26] Amvrazi E.G., Papadi-Psyllou A.T., Tsiropoulos N.G. (2010). Pesticide enrichment factors and matrix effects on the determination of multiclass pesticides in tomato samples by single-drop microextraction (SDME) coupled with gas chromatography and comparison study between SDME and acetone-partition extraction procedure. Int. J. Environ. Anal. Chem..

[27] Cengiz M.F., Başlar M., Basançelebi O., Kılıçlh M. (2018). Reduction of pesticide residues from tomatoes by low intensity electrical current and ultrasound applications. Food Chem..

[28] Graziela C.R., Andrade M., Monteiro S.H., Francisco J.G., Figueiredo L.A., Rocha A.A., Tornisielo V.L. (2015). Effects of types of washing and peeling in relation to pesticide residues in tomatoes. J. Braz. Chem. Soc..

[29] Bonnechère A., Hanot V., Bragard C., Bedoret T., van Loco J. (2012). Effect of household and industrial processing on levels of pesticide residues and degradation products in melons. Food Addit. Contam..

[30] Kontou S., Tsipi D., Oreopoulou V., Tzia C. (2001). Determination of ETU in tomatoes and tomato products by HPLC-PDA. Evaluation of cleanup procedures. J. Agric. Food Chem..

[31] Cengiz M.F., Certel M. (2014). Effects of chlorine, hydrogen peroxide, and ozone on the reduction of mancozeb residues on tomatoes. Turk. J. Agric. For..

[32] Keikotlhaile B.M., Spanoghe P., Stoytcheva M. (2011). Pesticide residues in fruits and vegetables. Pesticides—Formulations, Effects, Fate.

[33] Rasolonjatovo M.A., Cemek M., Cengiz M.F., Ortaç D., Büşra Konuk H., Karaman E., Kocaman A.T., Göneş S. (2017). Reduction of methomyl and acetamiprid residues from tomatoes after various household washing solutions. Int. J. Food Prop..

[34] Kwon H., Kim T.K., Hong S.M., Se E.K., Cho N.J., Kyung K.S. (2015). Effect of household processing on pesticide residues in field-sprayed tomatoes. Food Sci. Biotechnol..

[35] Al-Taher F., Chen Y., Wylie P., Cappozzo J. (2013). Reduction of pesticide residues in tomatoes and other produce. J. Food Prot..

[36] Anastasiades M., Lehotay S.J., Stajnbaher D., Schenck F.J. (2003). Fast and easy multiresidue method employing acetonitrile extraction/partitioning and dispersive solid-phase extraction for the determination of pesticide residues in produce. J AOAC Int..

[37] SANTE/12682/2019 Method Validation and Quality Control Procedures for Pesticide Residues Analysis in Food and Feed. https://ec.europa.eu/food/sites/food/files/plant/docs/pesticides_mrl_guidelines_wrkdoc_2019-12682.pdf.

[38] Shrivastava A., Gupta V.B. (2011). Methods for the determination of limit of detection and limit of quantitation of the analytical methods. Chron. Young Sci..

[39] Angioni A., Garau V.L., Aguilera A., Del Real M., Melis E.V., Minelli C., Tuberoso C., Cabras P. (2003). GC-ITMS determination and degradation of captan during winemaking. J. Agric. Food Chem..

[40] Rodrigues A.A.Z., De Queiroz M.E.L.R., De Oliveira A.F., Neves A.A., Heleno F.F., Zambolim L., Freitas J.F., Morais E.H.C. (2017). Pesticide residue removal in classic domestic processing of tomato and its effects on product quality. J. Environ. Sci. Health B.

[41] Reiler E., Jørs E., Bælum J., Huici O., Alvarez Caero M.M., Cedergreen N. (2015). The influence of tomato processing on residues of organochlorine and organophosphate insecticides and their dietary risk. Sci. Total Environ..

[42] Angioni A., Schirra M., Garau V.L., Melis M., Tuberoso C.I.G., Cabras P. (2004). Residues of azoxystrobin, fenhexamid and pyrimethanil in strawberry following field treatments and the effect of domestic washing. Food Addit. Contam..

[43] Tianxi Yang T., Doherty J., Zhao B., Kinchla A.J., Clark J.M., He L. (2017). Effectiveness of Commercial and Homemade Washing Agents in Removing Pesticide Residues on and in Apples. J. Agric. Food Chem..

[44] Commission Directive 2006/125/EC of 5 December 2006 on Processed Cereal-Based Foods and Baby Foods for Infants and Young Children. http://data.europa.eu/eli/dir/2006/125/oj.

[45] Commission Directive 2006/141/EC of 22 December 2006 on Infant Formulae and Follow-On Formulae and Amending Directive 1999/21/EC. http://data.europa.eu/eli/dir/2006/141/oj.

