# Supplementary material for: Effects of Industrial Processing on Pesticide Multiresidues Transfer from Raw Tomatoes to Processed Products
Source: Foods. 2020 Oct 19;9(10):1497. doi: 10.3390/foods9101497 (PMC7588992; doi:10.3390/foods9101497)
Supplement: Supplementary file 1 [file foods-09-01497-s001.pdf]

Table S1. Active ingredients, types, and LC-MS/MS-MRM m/z ions used for qualitative and quantitative analysis.

	Pesticide	Type of pesticide[¥]	t_R (min)	Precursor ion (m/z)	Product ions (m/z)
**1**	Cyromazine*	IGR	2.86	167	68
					85
2	Methamidophos	I	2.89	142	94
					125
3	Acephate	I	3.47	184	125
					143
4	Formetanate*	I/A	3.63	222	65
					165
5	Pymetrozine*	I	3.84	218	79
					105
6	Omethoate	I	3.84	214	109
					125
7	Propamocarb*	F	3.87	189	102
					74
8	Oxamyl*	N	4.45	237	72
					90
9	Methomyl*	I	4.77	163	88
					106
10	Flonicamid*	I	4.81	230	174
					203
11	Thiamethoxam*	I	4.90	292	181
					211
12	Carbendazim	F	5.00	192	132
					160
13	Monocrotophos	I	5.06	224	127
					193
14	Chlordimeform	A	5.15	197	117
					152
15	Cypermethrin*	I	5.52	433	89
					133
16	Imidacloprid*	I	5.55	256	128
					175
17	Methiocarb*	I	5.77	242	170
					185
18	Dimethoate*	I	6.04	230	79
					125
19	Acetamiprid*	I	6.05	223	56
					126
20	Cymoxanil*	F	6.51	199	110
					128
21	Thiacloprid*	I	6.64	253	90
					126
22	Atrazine-desethyl	H	6.68	188	68
					146
23	Aldicarb	I	7.30	213	89
					116

24	Pirimicarb*	I	8.05	239	72
					182
25	Dichlorvos	I	8.27	221	109
					127
26	Thiophanate-methyl*	F	8.40	343	93
					151
27	Metribuzin*	H	8.51	215	84
					187
28	Carbofuran	I	8.58	222	123
					165
29	Carbaryl	I	9.06	202	127
					145
30	Imazalil*	F	9.54	297	159
					201
31	Fosthiazate*	N	9.60	284	61
					227
32	Disulfoton-Sulfoxide	I	9.73	291	157
					185
33	Flutriafol*	F	10.49	302	95
34	Metalaxyl*	F	10.59	280	160
					220
35	Methidathion	I	10.71	303	145
36	Azinphos-methyl	I	10.95	318	132
					261
37	Chlorantraniliprole*	I	11.00	483	285
					453
38	Pyrimethanil*	F	11.01	200	82
					107
39	Azoxystrobin*	F	11.44	404	226
					329
40	Diethofencarb	F	11.45	268	124
					226
41	Propanil	H	11.61	218	127
					162
42	Fenamidone*	F	11.68	312	92
					236
43	Diclobutrazol	F	11.69	328	70
					159
44	Boscalid*	F	11.86	343	272
					307
45	Dimethomorph*	F	12.13	388	165
					301
46	Mandipropamid*	F	12.16	412	328
					356
47	Benthiavalicarb*	F	12.24	340	72
					180
48	Molinate	H	12.33	188	83
					126
49	Chloroxuron	H	12.45	291	125
					164

50	Myclobutanil*	F	12.48	289	70
					125
51	Bifenazate*	A	12.49	301	170
					198
52	Cyproconazole* 1	F	12.58	292	70
					125
53	Triadimenol*	F	12.64	296	70
					99
54	Iprovalicarb*	F	12.70	321	119
					186
55	Fenhexamid*	F	12.74	302	55
					97
56	Azinphos-ethyl	I	12.76	346	132
					55
57	Tetraconazole*	F	12.77	372	159
					108
58	Cyproconazole* 2	F	12.78	292	70
					125
59	Mepanipyrim*	F	12.80	224	77
					106
60	Spirotetramat*	I	12.80	374	270
					302
61	Flufenacet*	H	12.81	224	152
					194
62	Ethoprop*	I/N	12.91	243	97
					131
63	Bupirimate*	F	12.93	317	166
64	Cyazofamid*	F	12.97	325	108
					217
65	Flusilazole	F	13.24	316	165
					247
66	Cyprodinil*	F	13.27	226	65
					77
67	Fenamiphos*	I	13.27	304	217
					234
68	Iprodione*	F	13.41	330	245
69	Aclonifen*	H	13.51	265	182
					218
70	Penconazole*	F	13.65	284	70
					159
71	Tebuconazole*	F	13.76	308	70
					125
72	Napropamide*	H	13.82	272	58
					171
73	Benalaxyl*	F	13.96	326	91
					148
74	Spinosyn *A	I	14.14	732	98
					142
75	Zoxamide*	F	14.17	336	159
					187
76	Pyraclostrobin*	F	14.18	388	163

					194
77	Cyflufenamid	F	14.24	413	241
					295
78	Bitertanol	F	14.27	338	70
					100
79	Clofentezine*	A	14.28	303	102
					138
80	Phosalone	I/A	14.29	368	111
					182
81	Metrafenone*	F	14.46	409	209
					227
82	Difenconazole*	F	14.58	406	251
					337
83	Chlorpyrifos-methyl*	I	14.63	322	125
					290
84	Ametoctradin*	F	14.69	276	186
					191
85	Spinosyn* D	I	14.71	746	98
					142
86	Indoxacarb*	I	14.73	528	150
					203
87	Cycloate	H	14.78	216	83
					154
88	Hexaflumuron	IGR	14.81	461	141
					158
89	Trifloxystrobin*	F	14.82	409	150
					186
90	Quizalofop-ethyl*	H	15.32	373	255
					271
91	Cycloxydim*	H	15.37	326	101
					180
92	Buprofezin*	I	15.54	306	106
					201
93	Tebufenpyrad*	I/A	15.55	334	117
					145
94	Emamectin Benzoate*	I	15.59	887	126
					158
95	Propaquizafop*	H	15.61	444	299
					327
96	Metaflumizone*	I	15.63	507	116
					178
97	Oxadiazon*	H	15.73	362	220
98	Allethrin	I	15.77	303	103
					135
99	Piperonyl butoxide*	S	15.83	356	119
					177
100	Chlorpyriphos*	I	16.03	350	97
					198
101	Hexythiazox*	I/A	16.07	353	168
					228

102	Pyriproxyfen*	I	16.12	322	96
					185
103	Pendimethalin*	H	16.13	282	194
					212
104	Flufenoxuron	I	16.21	489	141
					158
105	Propargite	A	16.35	368	81
					231
106	Lufenuron*	I	16.53	510	141
					158
107	Etoxazole*	A	16.54	160	113
					141
108	Fenpyroximate(E)*	A	16.66	422	107
					366
109	Deltamethrin*	I/A	16.85	523	181
					281
110	Acrinathrin*	I/A	16.92	559	181
					208
111	Pyridaben*	I/A	17.09	365	147
					309
112	Tau Fluvalinate*	I/A	17.62	503	181
					208
113	Fenarimol	F	17.86	331	238
					313
114	Etofenprox*	I	17.88	394	107
					177
115	Bifenthrin	I	17.91	442	166
					181
116	Famoxadone*	F	18.84	392	93

* pesticide authorized on tomatoes

¥A: acaricide; F: fungicide; H: herbicide; I/A: acaricide/insecticide; I: insecticide; S: synergist a.i. IGR: insect growth regulator; I/N: insecticide/nematocide.
